# Draft genome sequence of an antibiotic-resistant *Heyndrickxia oleronia* strain UL23-01-03 isolated from the Arabian Sea coast, Dakshina Kannada, India

**DOI:** 10.1128/mra.00494-25

**Published:** 2025-06-10

**Authors:** Sanjana N. S., Vikas Balajirao Maske, Rekha Punchappady Devasya, Bhagwan Narayan Rekadwad

**Affiliations:** 1 MicrobeAI Lab, Division of Microbiology and Biotechnology, Yenepoya Research Centre, Yenepoya (Deemed to be University) 489734, Mangalore, Karnataka, India; 2 Packgene Biotech Inc., Houston, Texas, USA; 3 Division of Microbiology and Biotechnology, Yenepoya Research Centre, Yenepoya (Deemed to be University), Mangalore, Karnataka, India; California State University San Marcos, San Marcos, California, USA

**Keywords:** antibiotics, antibiotic resistance in the environment, coastal microbiome, *Heyndrickxia oleronia*, whole genome sequencing

## Abstract

Here, we present the draft genome of antibiotic-resistant *Heyndrickxia oleronia* UL23-01-03 isolated from the Arabian Sea coast, Mangalore, India. The draft genome consists of 53 contigs totaling 5,003,978 bp, with a guanine–cytosine content of 34.8% and an OrthoANI of 97.37% compared to the reference strain.

## ANNOUNCEMENT

*Heyndrickxia* spp. comprises a group of gram-positive, aerobic, rod-shaped bacteria with mesophilic and moderately thermophilic bacteria. The first representative type strain of *Heyndrickxia oleronia* was initially isolated from a termite’s hindgut from the island Île d'Oléron, France (1). Herein, we present the draft genome sequence of *H. oleronia* UL23-01-03. The crystalline-sandy soil core sample was collected from a depth of 20 cm from the Arabian Sea coast (12°47′25″ N, 74°50′27″ E), Mangalore, India. We isolated the antibiotic-resistant strain UL23-01-03 (on Luria-Bertani agar containing 8 mcg/mL meropenem at the following conditions: temperature, 37°C; pH 7.0; incubation time, 24–48 h; and at pH 7.0). Furthermore, antibiotic resistance tests (on media containing 6 mcg/mL penicillin, 10 mcg/mL ampicillin, and 30 mcg/mL amoxicillin), followed by sequencing and genomic analysis of strain UL23-01-03, helped us uncover antibiotic resistance genes and develop a strategy to prevent antimicrobial resistance.

The *H. oleronia* UL23-01-03 was cultivated on Luria-Bertani agar at the cultivation conditions mentioned earlier (Tables S1 and S2). Cell lysis of UL23-01-03 was achieved using 1× Tris-EDTA buffer with 20% SDS and 100 µg/mL proteinase K, and the genomic DNA was extracted using the phenol-chloroform-isoamyl (25:24:1) method (2–4). The purified genomic DNA was first sheared into smaller pieces using an E210 ultrasonicator (Covaris, Inc., USA) (5–7). DNA fragments of about 500 bp were cleaned up with Ampure beads (8) and measured with the Qubit dsDNA High Sensitivity assay kit (9–11). Paired-end libraries were prepared using the NEBNext Ultra II DNA Library Prep Kit and sequenced (2 × 150 bp chemistry) (12, 13) on an Illumina HiSeq 4000 (USA) (14). The sequencing generated 5,627,156 reads, which were filtered and checked for quality using Trimmomatic (v.0.39) (15). The *de novo* genome sequence assembly was prepared using SPAdes Genome Assembler (v.4.0.0), with a k-mer length of 127 (16). The resulting assembly had a consensus length of 5,003,978 bp spanning 53 contigs, with an *N*50 value of 304,960 bp and an *L*50 value of six contigs. The genome has an OrthoANI value of 97.37% as compared to the reference strain (H. oleronia J19TS1, GCF_018332875.1), which was calculated by OrthoANI using USEARCH (version OrthoANIu) (17).

Functional annotation using the National Center for Biotechnology Information Prokaryotic Genome Annotation Pipeline (v.6.8) (18–20) predicted 5,033 total genes, 4,924 coding sequence, 4,857 coding genes, 7 rRNA operons, 97 tRNAs, and five ncRNAs. The genome of *H. oleronia* UL23-01-03 was analyzed for antibiotic resistance genes using the DDBJ Fast Annotation and Submission Tool (DFAST) (21). DFAST (v.1.3.0) predicted the presence of Cfr 23S ribosomal RNA methyltransferase [cfr(B)], msr-type ABC-F protein [msr(G)], and elongation factor Tu (tufA) genes responsible for kirromycin resistance (22). The default parameters were used for all software.

## Data Availability

The draft genome sequence of *Heyndrickxia oleronia* UL23-01-03 has been submitted to the GenBank under accession number JBIMPO000000000, BioProject number PRJNA1175062, and BioSample identifier SAMN44355377. Raw sequence reads were deposited in the GenBank Sequence Read Archive under the accession number SRR31177223. The DFAST analysis data of UL23-01-03 and Tables S1 and S2 were deposited in Zenodo via links https://doi.org/10.5281/zenodo.14792487 and https://doi.org/10.5281/zenodo.14792458.
